# Role and Mechanism of Epigenetic Regulation in the Aging of Germ Cells: Prospects for Targeted Interventions

**DOI:** 10.14336/AD.2024.0126

**Published:** 2024-01-26

**Authors:** Xiang-Chun Huang, Yi-Nan Jiang, Hai-Juan Bao, Jie-Lin Wang, Rong-Jin Lin, Jing Yuan, Jing-Yuan Xian, Yang Zhao, Shuo Chen

**Affiliations:** ^1^Department of Obstetrics and Gynecology, Department of Gynecologic Oncology Research Office, Guangzhou Key Laboratory of Targeted Therapy for Gynecologic Oncology, The Third Affiliated Hospital of Guangzhou Medical University, Guangzhou, China.; ^2^Guangdong Provincial Key Laboratory of Major Obstetric Diseases, Guangdong Provincial Clinical Research Center for Obstetrics and Gynecology, Guangdong-Hong Kong-Macao Greater Bay Area Higher Education Joint Laboratory of Maternal-Fetal Medicine, The ThirdAffiliated Hospital of Guangzhou Medical University, Guangzhou, China.

**Keywords:** Epigenetics, Spermatozoa, Premature ovarian failure, Aging

## Abstract

In modern times, a notable trend toward delayed childbearing has been observed in most developed countries. As a result, sperm aging and quality loss, as well as premature ovarian failure (POF), have emerged as major causes of infertility. The pathogenesis of sperm aging and POF is complex and has not been clearly elucidated. However, evidence from some studies has linked germ cell aging to epigenetic modifications. Epigenetics refers to the heritable changes in gene expression that occur in the absence of any alterations to the gene’s nucleotide sequence. This paper systematically reviewed and analyzed the relevant literature to describe the relationship of DNA methylation, non-coding RNA regulation, histone modifications, chromatin remodeling, and RNA modifications with sperm aging and POF. In addition, we analyzed how sperm aging and POF can be mitigated via epigenetic interventions. This review could provide new therapeutic insights and guide strategies for improving sperm quality and ovarian function.

## 1. Introduction

The average age at which parents have their first child is rising annually due to several factors. These include the widespread use of effective contraception, advancements in assisted reproductive technologies, higher levels of women’s education and workforce participation, changes in societal values, increased gender equality, changes in partnership dynamics, housing challenges, economic instability, and the lack of supportive family policies [1].

**Figure 1.**
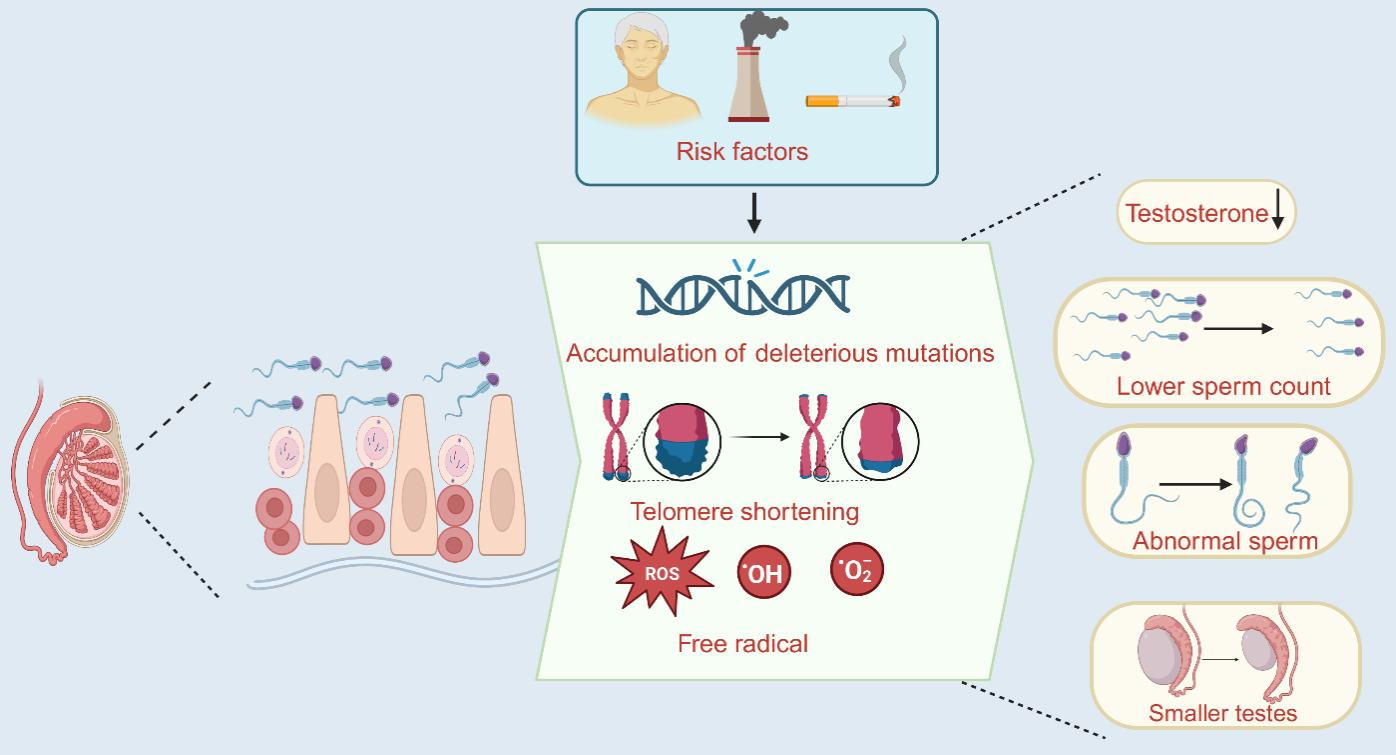
**Mechanisms of sperm senescence and its consequences**. Under the influence of various risk factors, accumulation of harmful mutations, shortening of telomere and formation of free radicals occur in sperm cells. Eventually sperm quality decreases (Created with BioRender.com).

Khandwala et al. analyzed the age of fathers of 168 867 480 live births in the United States over a 44-year period from 1972 to 2015, they found that the mean paternal age increased from 27.4 years-old in 1972 to 30.9 years-old in 2015 [2]. However, higher paternal age was linked to an increased risk of preterm labor, low birth weight, and low Apgar scores (a method of evaluating physiologic indicators and quality of life of organ systems of newborns at birth) [3]. There are strong indications that sperm quality declines with aging. This was evidenced by significantly lower testosterone levels, smaller testes, atrophy of the seminiferous tubules, lower sperm concentration and counts, increased sperm abnormalities, and decreased sexual activity [4-8]. The mechanisms of sperm senescence and the changes that occur in senescent spermatozoa are summarized in Figure 1.

In women, premature ovarian failure (POF) is also known as primary ovarian insufficiency (POI). It is characterized by elevated levels of gonadotropins (especially follicle-stimulating hormone) and irregular or absent menstruation before the age of 40. Today, POF is among the leading causes of female infertility. Women with POF experience a reduction in the initial number of primordial follicles, follicular apoptosis, or increased follicular destruction. In addition, their follicles may also fail to respond to gonadotropin stimulation [9]. Coulam et al. measured the age-specific prevalence of natural menopause in women in Rochester, Minnesota. The study showed that the prevalence of POF in women under 40 years of age was 1% [10].

**Figure 2.**
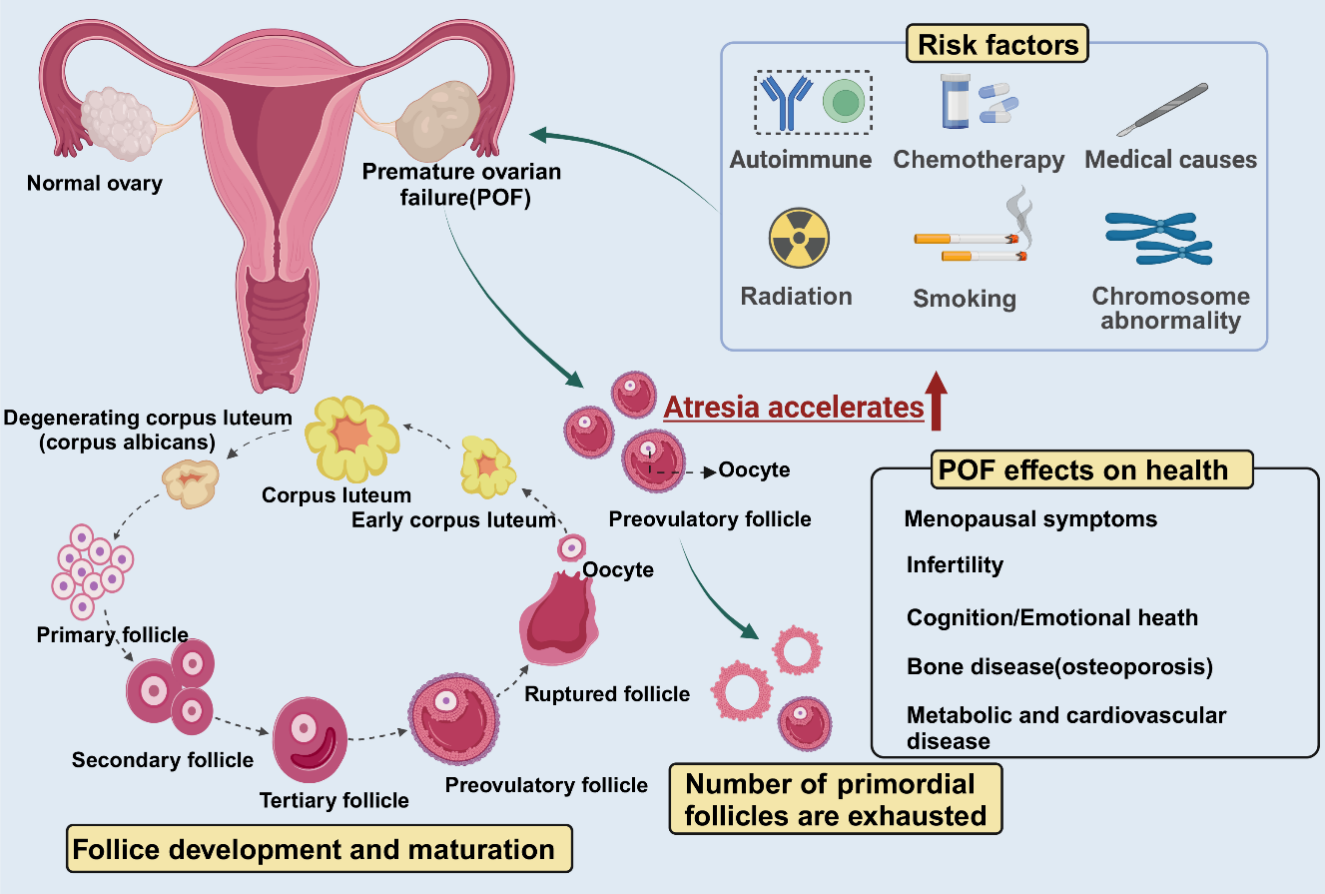
**Risk factors and consequences of POF and its effects on health**. Follicular atresia is accelerated in patients with premature ovarian failure due to factors such as autoimmunity and chemotherapy, which adversely affects a woman's physical and mental health (Created with BioRender.com).

The decreased expression of some POF genes, especially those critical for DNA repair, meiosis, and mitochondrial function, can contribute to ovarian aging [11]. Numerous studies also demonstrated that the causes of POF include genetic alterations such as chromosomal abnormalities and fragile pre-X chromosome mutations. Lifestyle factors, such as smoking, clinical factors, including autoimmune follicular destruction, chemotherapy, and radiation treatment, were also identified as contributors to POF in various research findings. [9, 12-21]. In many instances, POF results in infertility. In patients with ovarian failure due to partial ovariectomy or chemotherapy, the rates of ovulation and pregnancy were around 30.8% and 15.4%, respectively, compared to 5.0% and 1.7% in patients with secondary ovarian failure [22]. In addition to causing infertility, POF is also associated with a variety of health risks. These include severe menopause symptoms, decreased bone mineral density and an increased risk of fracture, early progression of cardiovascular disease, psychological distress including depression, anxiety, and decreased perceived psychosocial support, an early decline in cognitive performance, and dry eye [19, 23-25]. We summarized the causes of POF and its effects on women's health in Figure 2.

Cellular senescence refers to the gradual changes in a cell’s ability to proliferate and differentiate and the decline in its physiological functions over time. The accumulation of senescent cells leads to aging and the development of several disorders. Studies demonstrated the pathogenic role of senescent cells in a host of diseases. However, none of the currently known mechanistic pathways underlying cellular senescence are universal.

Epigenetics refers to the heritable changes in gene expression that occur in the absence of any alteration to the gene’s original nucleotide sequence. Several modes of epigenetic regulation were identified, with key mechanisms including DNA methylation, histone modifications, non-coding RNA (ncRNA) regulation, chromatin remodeling, and RNA modification. The rapid development of a range of technologies results in a corresponding increase in research on the relationship between epigenetics and germ cell aging. Accumulating evidence indicates that epigenetic changes play an increasingly important role in germ cell senescence. There is also compelling evidence suggesting that the predictive value of DNA methylation, histone modifications, ncRNA regulation, and RNA modifications in oocytes and spermatozoa may improve the diagnosis of male versus female infertility and enable targeted epigenetic interventions for reproductive disorders. In this context, we review the literature on age-related epigenetic changes in human and animal germ cells, focusing on their epigenetic regulation and discussing related therapies.

## 2. Regulatory mechanism of aging

**Figure 3.**
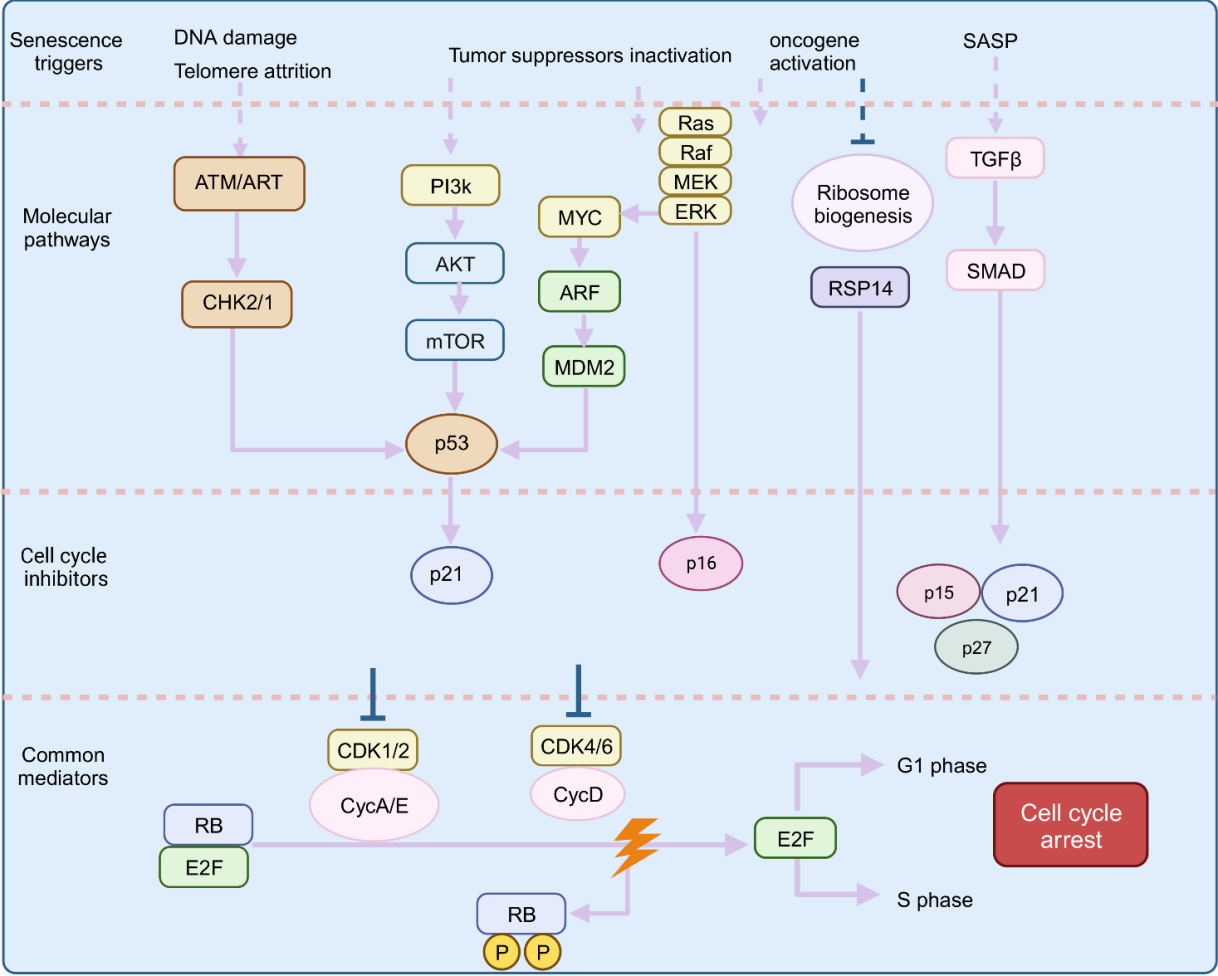
**The regulatory mechanism of cell aging**. Senescent cells exhibit DNA damage response (DDR), telomere shortening and damage, oncogene activation, and inactivation of tumor suppressors, which induce cell cycle arrest and inhibit cell proliferation through their corresponding signaling pathways (Created with BioRender.com).

Senescent cells exhibited the following phenomena: DNA damage response (DDR), telomere shortening and damage, oncogene activation, inactivation of tumor suppressors, and so on [26]. DNA damage results in the activation of ataxia-telangiectasia mutated proteins (ATM) and ataxia telangiectasia and Rad3-related (ATR) kinases and the upregulation of p53 [27]. The kinases ATM and ATR, located at the ends of unprotected chromosomes, are also activated after telomere shortening and damage, leading to p53 upregulation [28]. Oncogene-induced senescence via tumor suppressor genes involves three pathways: the Arf/p53/p21 pathway, p16/pRb pathway, and DDR pathway [29-32]. Oncogenes affect the formation of mature ribosomes and thereby disrupt protein synthesis, the cell cycle, and cell growth [33]. In addition, when oncogenes are activated, TGFβ secretion due to the senescence-associated secretory phenotype induces elevations in p15, p21, and p27 expression [34]. All these abovementioned pathways can inhibit cell cycle progression by blocking cyclin-dependent kinase (CDK) activity via the activation of CDK inhibitors, thereby causing CDK dysregulation. This induces cell-cycle arrest and inhibits cell proliferation. Figure 3 summarizes the signaling pathways associated with cellular senescence.

## 3. Epigenetic modifications

### 3.1 DNA methylation

DNA methylation, one of the earliest discovered and most common epigenetic modifications, can alter genetic expression without changing the DNA sequence. It is a chemical modification process in which specific bases on a DNA sequence acquire a methyl group. DNA methylation involves a chemical modification in which a methyl group is covalently linked to specific bases on the DNA sequence. This reaction is catalyzed by DNA methyltransferase (DNMT), with S-adenosylmethionine (SAM) serving as the methyl donor [35]. Specifically, DNA modification can regulate transcription in concert with other epigenetic modifications.

Altered DNA methylation signatures are a major hallmark of epigenetic recombination during germ cell aging, which lead to reduce gene transcription levels, alter chromatin, gene silencing, and hamper important developmental processes [36, 37]. The key enzymes involved in DNA methylation include DNMTs (DNMT1/3A/3B/3L) and Ten-Eleven-Translocation enzymes TETases (Tet1, Tet2, and Tet3) [35, 38]. These enzymes can directly affect the methylation level of the target gene and regulate its expression, and they play an important role in the degeneration of ovarian function. DNMTs are crucial for the development of mammalian, and their expression levels have been shown differences in spatiotemporal and subcellular. Meanwhile, Tet enzymes regulate DNA demethylation and transcription. Real-time DNA demethylation has been observed in several biological contexts, including tumorigenesis, embryonic stem cell maintenance, somatic recombination, and primordial germ cell development [39-41].

### 3.2 Non-coding RNA regulation

The ncRNA refers to RNA molecules that are not involved in coding proteins. This group of RNAs mainly consists of ribosomal RNA (rRNA), transfer RNA (tRNA), small nuclear RNA (snRNA), small nucleolar RNA (snoRNA), microRNA (miRNA), piwi-interacting RNA (piRNA), and circular RNA (circRNA). NcRNA regulation refers to the involvement of these RNA molecules in the regulation of gene expression and cellular functions through different mechanisms. Unlike mRNAs that encode proteins, ncRNAs are not translated into proteins but directly regulate biological processes within cells at the RNA level. Recent studies have shown that ncRNAs play essential roles in gene regulation, chromatin remodeling, cell differentiation, growth and development, and disease pathogenesis. Importantly, ncRNAs regulate gene expression via two primary mechanisms [42]. The first is gene silencing, wherein ncRNAs bind to mRNAs to form double strands, leading to mRNA degradation and translational inhibition. The second is post-transcriptional regulation, which involves the regulation of gene expression by ncRNAs at the post-transcriptional level. Further, ncRNAs can also induce chromatin modification, i.e., changes in chromatin configuration, by binding to specific proteins on the chromatin. These changes, in turn, regulate gene expression [43].

### 3.3 Histone modifications

Histone modification refers to chemical modifications that occur at specific sites on the histone molecule, including methylation, acetylation, ubiquitination, glycosylation, and so on. This leads to changes in chromatin structure and regulation of gene expression. In eukaryotic cells, DNA is packaged in the form of chromatin, with nucleosomes serving as the functional units. The core of a nucleosome comprises the globular regions of histone proteins, while the N-terminal tail protrudes from the nucleosome and can be subject to a range of post-translational modifications(PTMs), including acetylation, methylation, phosphorylation, and ubiquitylation[44, 45]. These modifications affect how DNA strands are packaged and alter their transcriptional activity.

**Figure 4.**
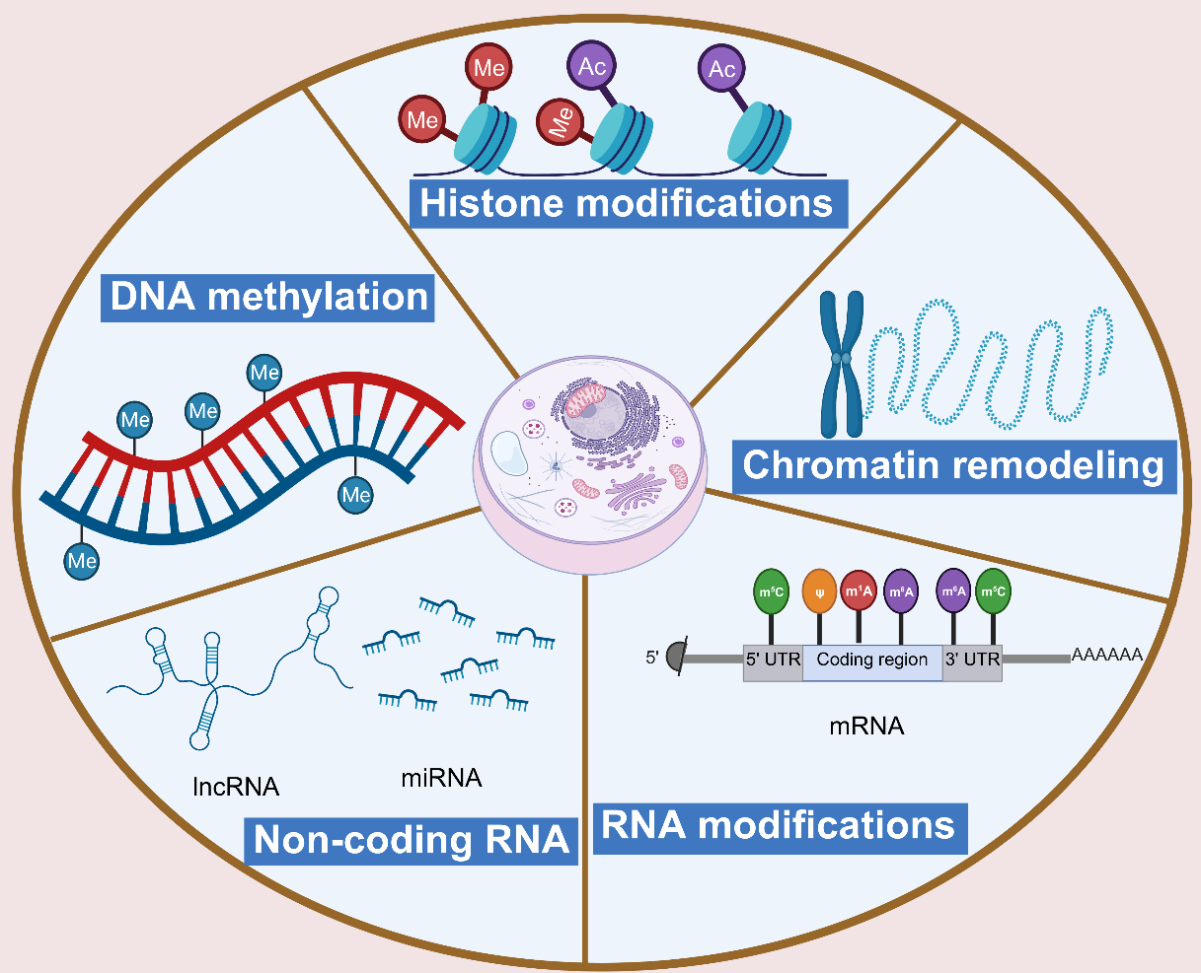
**Types of epigenetics**. The main mechanisms that cause epigenetic inheritance are DNA methylation, histone modifications, non-coding RNA regulation, chromatin remodeling, and RNA modifications (Created with BioRender.com).

### 3.4 Chromatin remodeling

Chromatin remodeling refers to the fact that the packaging state of chromatin, histones in nucleosomes, and corresponding DNA molecules are altered during processes such as replication and recombination of gene expression. In chromatin remodeling, the packaging configurations of chromatin, histones in nucleosomes, and corresponding DNA molecules are altered during processes such as replication and gene recombination. Chromatin remodeling complexes use adenosine triphosphate to reposition and modify the composition of nucleosomes across the genome and play a major role in controlling chromatin structure and regulating gene transcription[46]. When chromatin remodeling factors loosen the chromatin, the DNA becomes more accessible to RNA polymerase II, transcription factors, and other regulatory proteins, thereby initiating gene transcription. On the contrary, when the chromatin is dense, this accessibility is low, which inhibits the transcription of related genes.

### 3.5 RNA modifications

RNA modification is the process of chemical modification that occurs on an RNA molecule. The properties and functions of RNA are altered by introducing different chemical groups or structures on the nucleotides in the RNA molecule. RNA modification involves the base modification of RNA molecules, constituting the epitranscriptome. It has important biological functions and is involved in the onset and development of various human diseases. RNA modifications such as N6-methyladenosine(m6A), 5-methylcytidine(m5C), 1-methyladenosine(m1A), and N7-methylguanosine(m7G), among others, are widely distributed in messenger RNAs (mRNAs), transfer RNAs (tRNAs), ribosomal RNAs (rRNAs), small non-coding RNAs (sncRNAs), long non-coding RNAs (lncRNAs), and some other RNAs [47]. Their roles include the maintenance of mRNA stability, mRNA precursor splicing, polyadenylation, mRNA transport, and translation initiation [48]. The above epigenetic categories are summarized in Figure 4.

## 4. Epigenetic modifications and senescent spermatozoa

### 4.1 DNA methylation and senescent spermatozoa

Abnormal DNA methylation is a typical characteristic of in the process of mammalian aging. One of the hallmarks of aging tissues, including spermatozoa, is age-related changes in DNA cytosine methylation in cytosine-phosphate-guanine (CpG) dinucleotides. Research based on overall or local DNA methylation analysis revealed that spermatozoa exhibited age-related methylation changes. In one study, all but one donor exhibited age-related increases in mitotic methylation, such that the number of hypomethylated regions decreased significantly with age [49]. Moreover, evidence showed that sperm from older men had more hypermethylated than hypomethylated CpG sites [50]. Additionally, the level of DNA hypermethylation in the rDNA promoter region of sperm increased significantly with age [51].

Age-dependent DNA methylation was also observed in animal sperm. In one study, 5283 out of the 5319 (99.3%) differentially methylated regions (DMRs) in sperm cells showed higher levels of DNA methylation in mature rats than in young rats, with only 36 (0.7%) DMRs exhibiting lower methylation. The average methylation of individual CpG dinucleotides also increased in an age-dependent manner across genomic landscapes, namely, coding regions, promoters, and intergenic regions [52]. Another study in Japanese black cattle confirmed that eight out of nine CpG sites (CpG-1, CpG-A1-A8) showed an age-dependent increase in methylation levels [53]. In contrast, another study in young and old mice showed that aging induced the hypomethylation of sperm chromosomal DNA [54]. Age-related changes in DNA methylation were summarized in Table 1.

Age-related sperm DMRs are not randomly distributed throughout the genome, but are instead clustered at certain chromosomal locations [55]. For example, chromosome 19 showed a two-fold enrichment of age-related sperm DMRs, but the orthologous marmoset chromosome 22 did not appear to exhibit age-related changes in DNA methylation [56]. Moreover, hypermethylated CpG sites usually shifted toward the distal region of the gene with age, while hypomethylated sites typically occurred closer to the transcription start site [50].

Increased levels of methylation, both overall and within individual genes, can impact sperm morphology, counts, and motility. Aging may reduce male fertility by altering DNA methylation in sperm [57, 58]. Alterations in overall DNA methylation affected protein expression levels in sperm, leading to abnormalities in human semen [59]. Excessive levels of methylation in spermatozoa led to significant reductions in sperm count, total viability, and non-progressive motility [8]. In terms of individual genes, the methylation of *MEST*, *GNAS*, and the repeat element *LINE1* was significantly correlated with sperm counts [60]. Significant differences in *H19* and *Gtl2* methylation levels were also observed between infertile patients and fertile controls [61, 62].

The effects of increased paternal age on sperm methylation can affect the behavior and neurodevelopment of the offspring [56]. A recent study showed that spermatozoa from older fathers exhibit significant methylation abnormalities in genes associated with autism spectrum disorder, schizophrenia, and bipolar disorder [55].

The aforementioned studies indicate alterations in DNA methylation levels within spermatozoa as age progresses. The observed methylation patterns differ across various studies, potentially due to factors like species diversity, methods employed for sample analysis, and selection of genomic regions for investigation. Furthermore, the impact of age on sperm DNA methylation has been associated with male fertility, sperm morphology, and the development of genetic disorders.

**Table 1:** Changes in DNA methylation, ncRNA and histone modification levels during sperm aging.

Epigenetic modification	Epigenetic levels in elderly males	Species	References
**Overall DNA methylation**	Increase	Human	[49]
**CpG site methylation**	More hypermethylated than hypomethylated CpG sites	Human	[50]
**rDNA promoter region methylation**	Increase	Human	[51]
**DNA methylation**	5283 (99.3%) DMRs increased, 36 (0.7%) DMRs decreased	Mouse	[52]
**DNA methylation**	Eight out of nine CpG sites show an increase in methylation levels	Cattle	[53]
**Ovall DNA methylation**	Decrease	Mouse	[54]
**rRNA and lncRNA**	Decrease	Rat	[63]
**tRNAs, precursor miRNAs, and miRNAs**	Increase	Rat	[63]
**miRNAs and tsRNAs**	DMRs exist at different ages	Bull	[64]
**miR-125a-5p**	Increase	Human	[65]
**microRNAs and piRNAs**	Differential expression of 184 microRNAs and 227 piRNAs	Mouse	[66]
**lncRNA**	Differential expression of 715 lncRNAs	Mouse	[68]
**tSNA**	34 up-regulated and 11 down-regulated tRFs and tiRNAs	Mouse	[67]
**miR-574**	Increase	Human	[69, 70]
**H3K27me3**	Increase	Human	[66, 80]
**H3K9me3 and H3K4me2**	Decrease	Human	[80]
**H3K27ac**	Depends on the stage of aging testicles		
**H3K27me2/3**	Increase		

### 4.2 Non-coding RNA and senescent spermatozoa

As men age, the expression levels of various RNAs change in spermatocytes. In rat sperm, the fraction of rRNAs and lncRNAs decreased with age, while that of tRNAs, precursor miRNAs, and miRNAs increased with age [63]. A study of four bulls revealed differential expressions of miRNA and tRNA-derived small RNA (tsRNA) between bulls of different age groups. These differentially expressed miRNAs were involved in embryonic metabolism and protein synthesis [64]. Other studies reported ncRNA changes in aging spermatozoa. For example, miR-125a-5p was upregulated in the spermatozoa of aging men [65]. Moreover, the differential expression of 184 miRNAs and 227 piRNAs was observed between young and old mice [66]. Moreover, altered levels of lncRNAs and tsRNAs were detected in the testes of mice from different age groups, indicating that these levels are altered during testicular aging [67, 68]. The ncRNA content of spermatozoa changed significantly with age and was summarized in Table 1.

The ncRNAs expressed in the spermatozoa of aging men regulate sperm status through different pathways. In spermatozoa from older males, miR-574 was upregulated and inhibited mitochondrial function by targeting mt-ND5. Moreover, upregulated miR-23a/b-3p bound to the 3' untranslated regions (UTRs) of the *ODF2* and *UBQLN3* genes to downregulate their expression. These changes decreased sperm count and viability and lead to the production of morphologically abnormal spermatozoa [69, 70]. Long intergenic ncRNA (lincRNA) deletions also reduced fertility in male *C. elegans* nematodes [71]. Interestingly, other studies showed that age-related differentially expressed piRNAs in spermatozoa are mainly involved in gene expression, transcription, protein modification, and cell development during spermatogenesis and testicular development [63, 72].

As with DNA methylation, changes in ncRNA expression can also affect the health of offspring. The upregulated tRNA-derived fragment (tRF) and tiRNA-targeted genes in aged male mice were implicated in neurogenesis and nervous system development. Moreover, the injection of sperm tsRNAs from aged male mice into congeners was found to induce anxiety-like behaviors in F1 male mice [67].

As men age, alterations occur in RNA expression levels within sperm cells, encompassing diverse RNA categories like miRNAs, tRNAs, and lncRNAs. The expression of ncRNA in aging sperm regulates sperm characteristics, influencing sperm count, viability, morphology, and contributes to processes in neurodevelopment and cellular development. These discoveries suggest a potential association between changes in RNA expression and the health as well as behavioral characteristics of offspring.

### 4.3 Histone modifications and senescent spermatozoa

Histones play important roles in sperm formation. For example, the top H3K4me3-rich genes were involved in sperm tail formation, chromatin condensation, and sperm function [73]. Bovine H4 acetylation was important for spermatogenesis, early embryo implantation, and sperm capacitation [74]. Meanwhile, decreased H4 acetylation and increased levels of H3 modification resulted in sperm aberrations [75]. Reduced H4 acetylation was detected in both asthenoteratozoospermic and asthenozoospermic samples [75]. There also existed a negative correlation between H3K4me2 and sperm concentration, motility, and mitochondrial activity [76].

Abnormalities in the regulatory units of histones can also lead to decreased sperm quality. In elongating spermatids, the deficiency of DOT1L (a histone methyltransferase) resulted in the incomplete replacement of histones, leading to abnormal protein recruitment. This induced reduced testicular weight, sperm abnormalities, and male sterility [77, 78]. Similarly, mice in which lysine-specific histone demethylase 2 (*Kdm2a*) was knocked out or mutated exhibit significant reductions in the total number of spermatozoa and the density of seminiferous tubules, as well as decreased sperm motility [79].

Aging induces changes in histone modifications. A study found that in older fathers, a small region on chromosome 5 contained 90% of the differential histone PTMs, corresponding to altered H3K27me3 signals. Most of the genes in this region belonged to the spermatogenesis-related gene family (Speer) [66]. Another study found that H3K9me3 and H3K4me2 signals were reduced in male germline cells from old testes. In contrast, H3K27me2/3 signals were increased in senescent testicular germ line cells. Interestingly, the findings indicated that the intensity of H3K27ac can be lower or higher depending on the stage of the senescent testis [80]. We summarized the age-related changes in histone modification levels in sperm in Table 1.

Unfortunately, the relationship between histone modifications and spermatozoa function has been limitedly reported. Only specific histone PTMs have been identified in abnormal and senescent spermatozoa, and the full extent of changes in histone PTM profiles is unclear. Hence, we advocate for intensified and comprehensive research into the interplay between histones, their modifications, and sperm cells, aiming to delve deeper into intricate details and comprehend their significance in reproductive health. Such endeavors will aid in bridging existing knowledge gaps and advancing the landscape of reproductive medicine.

### 4.4 Chromatin remodeling and senescent spermatozoa

Defects in chromatin remodeling-related genes and proteins can cause sperm abnormalities, leading to male reproductive disorders. Male mice with *Rimklb* gene defects and *Ddx43* knockout (KO) exhibited lower chromatin aggregation and impaired histone-to-protamine exchange, resulting in decreased fertility [81, 82]. The deficiency of nuclear channel protein 210 like (NUP210L) protein might prevent adequate nuclear compaction by blocking the entry of histone variants/transition proteins/protamine into the nucleus and/or by inhibiting adequate core histone substitution. This induced abnormal sperm [83]. Reduced activity of the RNF8 and ubH2A pathways in spermatozoa from *L3MBTL2* conditional knockout (cKO) mice decreased protamine 1 deposition and chromatin condensation. This caused an increase in abnormal spermatozoa, a progressive decrease in sperm count, and premature testicular failure [84]. Male mice with abnormalities in Cecr2 (null allele, Cecr2Del, and hypomorph allele, Cecr2GT) — a chromatin remodeling protein — showed lower sperm counts and poorer sperm viability[85]. The regulatory mechanisms and roles of genes or proteins associated with chromatin remodeling in senescent spermatozoa were summarized in Table 2. Disruptions in chromatin remodeling-related genes or proteins could potentially impair sperm parameters, resulting in male reproductive disorders. Studies involving mice with experimentally manipulated genes showcased diminished sperm quality, irregular testicular function, and decreased sperm counts. Yet, investigations into the association between chromatin remodeling and aging remain limited, warranting further exploration in this domain.

### 4.5 RNA modifications and senescent spermatozoa

Studies on the relationship between RNA modifications and sperm aging are rather limited. However, an increase in paternal age was usually accompanied by a decrease in sperm quality, leading to infertility, which was characterized by decreased sperm counts and motility, increases in malformed sperm, and increased sperm necrosis [86]. Therefore, we speculated that aging leads to RNA modifications similar to those found in other forms of epigenetic regulation.

A recent study showed that the levels of m1A, Am, m6A, Cm, m7G, and Gm in total sperm RNA were significantly increased under conditions of azoospermia, while the expression of m5C and m^2^_2_^7^G was significantly increased in teratozoospermia [87].

The m6A is currently the most studied RNA modification. Specifically, m6A modification affected mRNA expression and was strongly associated with sperm motility, apoptosis, and metabolism [88]. Treatment with perfluoroheptanoic acid (PFHpA) to alter the levels of seven m6A-associated enzymes [89] and the direct knockdown of m6A regulators to reduce the levels of m6A RNA methylation in testicular tissues [90] both resulted in sperm dysfunction and led to infertility. Correspondingly, increased overall levels of m6A RNA modifications also resulted in testicular damage, causing male reproductive disorders [91].

**Table 2:** The regulatory mechanisms and effects of genes or proteins related to chromatin remodeling in aging sperm.

Genes or proteins related to chromatin remodeling	Regulatory mechanisms	Effects	References
**Ddx43**	Impairing exchange between histones and protamine	Low male fertility	[81]
**Rimklb**	Impairing exchange between histones and protamine	Low male fertility	[82]
**NUP210L**	Prevent histone variants/transition proteins/protamine from entering the nucleus and/or by preventing sufficient substitution of core histones	Abnormal sperm parameters	[83]
**L3MBTL2**	Reducing levels of RNF8 and ubH2A pathways leading to reduced deposition of protamine 1	Abnormal sperm parameters	[84]
**Cecr2**	387 differentially expressed genes	Abnormal sperm parameters	[85]

Wilms tumor 1-associated protein (WTAP) is a member of the m6A methyl writing complex (Writer) family. The deletion of this protein in WTAP-sKO mice resulted in reduced sperm counts and an infertility phenotype [92]. Meanwhile, the overexpression of *ALKBH5*, an “eraser” for m6A, promoted the G1-to-S phase transition in goat spermatogonia stem cells (SSCs) and inhibited apoptosis [66]. In contrast, *ALKBH5*-KO mice showed reduced sperm counts and impaired sperm viability and morphology, and differential gene expression analysis indicated that the genes downregulated in these mice were all associated with fertilization and reproduction [93, 94]. The m6A reader *YTHDC2* was highly expressed in spermatogenic cells, and its knockdown in mouse germ cells resulted in sperm defects and a loss of fertilization capacity [95, 96]. Meanwhile, LARP7 could mediate the 2'-O-methylation of U6 snRNA as an activator of box C/D snoRNP. And precursor mRNA splicing was dysregulated in male germ cells from LARP7-cKO mice, resulting in spermatogenic failure [97]. The types of RNA modifications in poor quality spermatozoa and the expression levels and effects of the associated proteins were summarized in Table 3.

Research concerning the correlation between RNA modifications and sperm aging remains scarce. Nonetheless, studies have investigated diverse RNA modification types and their impact on sperm function. These investigations underscore the significance of RNA alterations, notably m6A, in governing sperm function and fertility, hinting at their potential as a pivotal element in sperm aging.

### 4.6 Epigenetic modification-based treatment for senescent spermatozoa

Epigenetic changes in sperm can be reversed by a variety of methods, which have the potential to mitigate the age-induced decline in sperm quality. A recent study demonstrated a decrease in upregulated DNA methylation levels and a significant elevation in proliferating cell nuclear antigen expression after the transfusion of plasma from young animals to an older group. Moreover, the counts of spermatogonia and spermatocytes increased, similar to those in their younger counterparts [98]. Melatonin activated the PI3K/AKT signaling pathway and thereby increased sperm motility by promoting KIAA 1429-mediated m6A deposition [99]. A study showed that Zn supplementation could restore normal DNA methylation, spermatidylation, and seminiferous tubule in male gonads. Additionally, it could maintain SSCs, significantly reduced the mean percentage of ubiquitination and DNA breaks in sperm, as well as improved chromatin integrity, testicular organization, and spermatogenesis [100]. A traditional Chinese compound medicine called New Wenshen Shengjing Decoction was found to significantly reduce the level of H3K4me3 in mouse spermatozoa, thereby enhancing fertility [101]. Interestingly, the bacterium *Lactobacillus rhamnosus* reversed the increased miRNA (miR-155-5p, miR-26a-5p, miR-21-5p, miR-200c-3p, and miR-let7a-5p) and *DNMT1* expression in spermatozoa induced by a high-fat diet [102]. Yoga therapy also appeared to improve sperm quality by altering DNA methylation [103]. We summarized in Table 4 the therapeutic mechanisms and effects of reagents or methods for treating aging spermatozoa.

**Table 3:** Expression levels and effects of RNA modification types and related proteins in poor quality sperm.

Types of modifications or related proteins	Expression level	Effects	References
**m1A, Am, m6A, Cm, m7G and Gm**	Increase	Azoospermia	[87]
**m5C and m^2^_2_^7^G**	Increase	Teratozoospermia	[87]
**m6A**	Decrease	Sperm dysfunction and infertility	[89, 90]
**m6A**	Increase	Testicular injury	[91]
**WTAP**	Knockout	Reducing sperm concentration	[92]
**ALKBH5**	Knockout	Reducing sperm count, impaired sperm motility and morphology	[93, 94]
**YTHDC2**	Knockout	Sperm defects and loss of fertilization ability	[95, 96]

Methods for reversing epigenetic changes in sperm cover a variety of pathways, which offer hope for mitigating sperm quality decline due to aging. More research and in-depth exploration of the underlying mechanisms of these approaches will help us to better understand and cope with the problem of declining sperm quality.

## 5. Epigenetic modifications and POF

### 5.1 DNA methylation and POF

Similar to sperm aging, ovary senescence is associated with altered DNA methylation profiles. Liu et al. reported that the failure of GTCG methylation may lead to apoptosis and follicular atresia, a phenomenon in which follicles failed to continue to mature and eventually degenerated and disappeared after reaching a certain stage of development due to various reasons. Yu et al. reported that gene expression was lower in elderly women with a natural age-related decline of ovarian function, which was associated with higher genomic methylation [104]. Marshall et al. uncovered higher DNA methylation and histone H3K9me2 levels in oocytes of female mice aged 69-70 weeks than in oocytes of female mice aged 10-13 weeks [105]. In addition, Xi et al. revealed 422 DMRs between old and young pigs, and the genes with differential methylation and expression were found to be involved in the ovarian aging cycle [106].

**Table 4:** The therapeutic mechanism and effectiveness of reagents or methods for treating aging sperm.

Treatment reagents or methods	Therapeutic mechanism	Treatment effect	References
**Young plasma**	Downregulating methylation levels	Increasing count of spermatogonia and spermatocytes in elderly animals	[98]
**Melatonin**	Enhancing KIAA1429 expression and m6A RNA methylation to activate downstream PI3K/AKT signaling pathways	Improving the vitality and proliferation rate of GC-1 spg cells, and inhibiting cell apoptosis	[99]
**Zn**	Restoring normal DNA methylation	Improving chromatin integrity, testicular tissue, and spermatogenesis	[100]
**New Wenshen Shengjing Decoction**	Reducing H3K4me3 levels in sperm	Inhibiting embryonic cell apoptosis to promote early embryonic development	[101]
**Lactobacillus rhamnosus**	Reversing the expression of miRNA (miR-155-5p, miR-26a-5p, miR-21-5p, miR-200c-3p, and miR-let7a-5p) and DNMT1	Improving the survival rate of F0 animal offspring	[102]
**Yoga therapy**	Altering DNA methylation	Improving progressive sperm motility	[103]

DNA methylation regulates some genes related to ovarian function, and epigenetic alterations in these genes may cause ovarian dysfunction. Anti-Müllerian hormone (*AMH*) is a guanine-cytosine (GC)-rich gene, which can serve as a biomarker of diminished ovarian reserve. Some studies have shown that altered DNA methylation within the promoters of steroid hormone genes reduced their expression, ultimately leading to decreased ovarian steroid hormone secretion, reduced granulocyte viability, and premature follicular depletion [107-109]. Li et al. found that the methylation of the autophagy genes *Atg5* and *Lc3B* resulted in decreased autophagic activity, decreasing ovarian function in aging rats [110]. Meanwhile, Liu et al. confirmed that the deletion of *Rps26* in oocytes led to higher levels of DNA methylation at 5-cytosine, causing delayed oocyte development, cell death, and follicular atresia[111]. Additionally, Olse et al. found 4,199 CpG sites that are differentially methylated in wall granulosa cells (GCs) between women with a diminished ovarian reserve and normal controls [112].

DNMTs and TETases play an important role in the worsening of ovarian function. The mRNA and protein expression of the *Dnmt1*, *Dnmt3a*, and *Dnmt3l* genes and overall DNA methylation levels in the ovaries progressively decreased with age, whereas the expression of *Dnmt3b* progressively increased [110, 113]. Yue et al. discovered that the reduced expression of DNMT and alterations in whole-gene methylation in oocytes might be associated with decreased procreative potential in aged female mouse [114]. Tet1 was required to maintain oocyte number, oocyte quality, and follicular reserve. Thus, Tet1 deficiency promoted DNA demethylation in primordial germ cells, resulting in the decreased expression of meiotic genes. This, in turn, led to abnormal meiotic division and a reduced follicle reserve, which was closely related to POF [115, 116].

Ovarian aging is closely linked to alterations in DNA methylation, characterized by increased DNA methylation in oocytes and diminished expression of associated genes. These epigenetic variations might impair ovarian function, influencing both ovarian reserve and reproductive potential. However, further research is required to deeply investigate the mechanisms underlying these changes and their precise contribution to the process of ovarian aging.

### 5.2 Non-coding RNA and POF

Similar to sperm with decreased motility, senescent ovaries have different levels of ncRNAs than normal ovaries. Senescence affects the abundance of miRNAs involved in oocyte development. For example, miR-19b was highly enriched in the follicular fluid (FF), GCs of young cows and regulates genes associated with FoxO signaling, endocytosis, and NR3C1 in GCs [117]. Differentially expressed genes and miRNA interacted in young and senescent mice, which influenced folliculogenesis, oocyte growth and steroidogenesis [118]. It was shown that the expression of miR-23a and miR-27a was significantly higher in aged rats than in controls [119]. Increased miRNA143 and miRNA145 expression was also detected in the FF of women with reduced ovarian reserve [120]. Moreover, evidence showed that miR-27B, miR-151, miR-672, miR-26B, miR-92a, and other miRNAs could contribute to POF by inducing the apoptosis of ovarian GCs [121-123]. Similarly, miR-29a, miR-144, miR-190, miR-378, miR-146aC>G, miR-196a2T>C, and other miRNAs could contribute to POF by influencing follicle formation [115, 124, 125].

In a POF cell model (KGN cells), upregulated miR-497-3p was found to inactivate the PI3K/AKT/mTOR signaling pathway and promote DNA damage and apoptosis [126]. It was shown that miR-143-3p hindered steroid hormone synthesis by targeting the ubiquitin-conjugating enzyme E2 E3 (*Ube2e3*) and the luteinizing hormone and human chorionic gonadotropin receptor (*LHCGR*) [127]. High fat and high sugar consumption activated the Dab2ip/Ask1/p38-Mapk signaling pathway and promoted γH2A.X phosphorylation by inhibiting the expression of endogenous miR-146b-5p, leading to GCs senescence and POF [128]. The lncRNA BBOX1 antisense RNA 1 (BBOX1-AS1), which promoted granulocyte apoptosis through the over-absorption of miR-146b, induced POF [129]. Meanwhile, the lncRNA DLEU1 directly interacted with miR-146b-5p, and its overexpression sponged off miR-146b-5p, thereby increasing KGN cell apoptosis [130]. A study in older women identified a ceRNA network consisting of miRNAs binding to 11 differentially expressed lncRNAs and their mRNA targets, and this network was found to be involved in the regulation of the PI3K-Akt, FOXO, and p53 signaling pathways [131]. In addition, studies showed that the downregulation of *SAV1* via lncRNA/FMR6 binding promoted KGN cell apoptosis and inhibited cell proliferation, thus accelerating POF progression [132]. In addition, the circRNAs that showed altered expression during aging were mainly involved in metabolic processes, the regulation of secretory pathways, redox processes, steroid hormone biosynthesis, and insulin secretion pathways [133]. Table 5 summarized the expression levels and regulatory mechanisms of ncRNA in POF.

The latest research underscored the pivotal roles of miRNAs and lncRNAs in ovarian aging and POF, influencing follicular development, apoptosis, and hormone synthesis. Investigating these ncRNAs and their regulatory mechanisms further holds promise for unveiling novel therapeutic approaches to address ovarian dysfunction.

### 5.3 Histone modifications and POF

So far, numerous studies confirmed that histone modification not only played a crucial role in the process of normal aging but also actively participated in mammalian oocyte meiosis and activation. Although these studies did not directly prove the association between histone modifications and POF, they strongly suggested that histone modifications may significantly contribute to the development of POF [134]. Age-related declines in oocyte capacity were a major contributor to female infertility among mammals, and changes in histone modification were among the key factors associated with this infertility [135-138]. Yu et al. found that the levels of H3K4me1/2/3 in oocytes of young mouse were higher than those in senescent mouse oocytes. Furthermore, the expression level of Kdm1a, which was negatively correlated with H3K4me2 levels, increased in the oocytes in older genital sacs. Bui et al. found that the meiosis of pig oocytes was closely related to the phosphorylation level of histone H3 [139]. Suo et al. demonstrated that the level of histone acetylation in mouse oocytes also increased during aging. When histone acetylation was intentionally enhanced, most oocytes failed to form prokaryotic nuclei or form morphologically abnormal prokaryotic nuclei [140].

**Table 5:** The expression level and regulatory mechanism of ncRNA in POF.

ncRNA name	Expression level	Regulation mechanism	References
**miR-497-3p**	Increase	Deactivating the PI3K/AKT/mTOR signaling pathway	[126]
**miR-143-3p**	Increase	Targeting ubiquitin binding enzyme E2 E3 (Ube2e3), luteinizing hormone, and human chorionic gonadotropin receptor (LHCGR) to inhibit steroid hormone synthesis	[127]
**miR-146b-5p**	Decrease	Upregulation of ncRNA DLEU1 promotes apoptosis	[128]
**BBOX1-AS1**	Increase	Excessively absorbing miR-146b, increasing apoptosis of OGCs	[129]
**lncRNA DLEU1**	Increase	Absorbing MiR-146b-5p, increasing apoptosis of KGN cells	[130]
**lncRNA/FMR6**	Increase	Lowering SAV1, promoting apoptosis and inhibiting proliferation of KGN cells	[132]
**CircRNA**	194 CircRNAs upregulated and 207 CircRNAs downregulated	Participating in circDDX10-miR-1301-3p/miR-4660-SIRT3 axis	[133]

The anaphase-promoting complex initiated the transition from mid to late oocyte maturation by inducing the degradation of the cell cycle proteins B and securin [141]. In addition, the protein ubiquitin (Ub) E3 ligase also triggered the degradation of specific proteins, thus playing an important role in meiotic and mitotic processes [142]. Members of the family of E3 ligase Cullin ring finger ubiquitin ligase 4 (CRL4) interaction partners DCAF13 could involve in oocyte meiosis by adjusting the coupling activated protein [143]. Loss of DCAF13 in oocytes led to impaired meiotic process, defective chromatin condensation, polyubiquitination and PTEN degradation [144, 145]. In addition, Herbert et al. reported that the ubiquitination-mediated degradation of CCNB1 and securing was essential for mid- to paracrine transition during the meiotic maturation of oocytes. In addition, the deubiquitinase UCHIL3 and the ubiquitin-proteasome system was also implicated in follicular development [146, 147]. The aberrant histone modification of AcH4K12 disrupted the meiotic maturation process by causing abnormal spindle structure and mitochondrial dysfunction in oocytes. These modifications induced DNA damage and trigger early apoptosis[148], further suggesting that the histone ubiquitination/deubiquitination system played a vital role in follicular development and the maintenance of ovarian function, affecting meiosis [149]. Impaired meiosis might lead to meiotic arrest and degeneration of the oocyte and ultimately the ovary[150, 151].

Two main types of glycosylation affected a wide range of properties in intracellular proteins —O-linked glycosylation and N-linked glycosylation [152]. N-glycosylation of proteins in oocytes was a key cause of female fertility, and missense mutations in DPAGT1(an enzyme involved in N-glycosylation) led to female subfertility by hindering the development of follicles and the process of ovulation [152]. Protein O-glycosylation was involved in the regulation of female fertility by affecting the quality of oocytes generated.

SUMOylation was mediated by SUMO proteins, which regulated oocyte maturation and affected the function of mature oocytes by participating in processes such as oocyte meiosis and spindle formation [153-156]. Enzymes related to the SUMO protein function such as UBE21 (combined type E2 enzyme) could also through influencing the full play of the SUMO protein function involved in regulating the of oocytes [153]. In addition, some proteins that affected oocyte maturation were also involved in SUMOylation or SUMOylation, such as SENP2 (SUMO-specific peptidase 2) [157], septin (a GTP-binding protein), MAD3/Bub1b (BUBR1) (spindle assembly protein) and others [158, 159]. These findings indicated histone SUMOylation played an important role in oogenesis and ovarian function.

Changes in histone modifications and the upregulation of genes associated with premature aging were observed in natural human placental mesenchymal stromal cells (hPMSC) when compared to frozen hPMSC[160]. The genetic factors contributing to POF were currently under extensive investigation, encompassing chromosomal abnormalities and mutations in the fragile X syndrome-related gene (Fmr1). Mutations in the Fmr1 gene led to high methylation of H3K9 and low methylation of H3K4, resulting in an overall decrease in histone acetylation. Under typical conditions, histones H3 and H4 should exhibit high acetylation levels, while H3K4 should show high methylation, and H3K9 should remain hypomethylated[161, 162].

To sum up, histone modifications way through multiple aspects affects the growth and development process of reproductive cells. However, although there is no direct evidence that there is a definite relation between histone modification and POF, the inability of follicles to develop and mature properly causing by any subtle changes during follicular development could lead to follicular atresia. This could result in a decrease in the overall follicular pool and a decline in ovarian function, ultimately progressing to POF or accelerated ovarian senescence [150, 163, 164]. This affects a woman's reproductive health, which is the direction of our further research to explore in the future.

### 5.4 RNA modifications and POF

The protein levels of fat mass and obesity-associated protein (FTO), an m6A-demethylating enzyme, were found to decrease with age. Moreover, the total m6A levels increased in an age-dependent manner [165, 166]. FTO could bind to the *FOS* mRNA 3'UTR. Hence, FTO knockdown delayed *FOS* mRNA degradation, upregulating FOS expression in GCs and ultimately inducing GC-mediated ovarian senescence [167].

A significant decrease in total m6A levels was observed in a POF rat model induced by 4-vinyl cyclohexene epoxide. Further, alterations in *ALKBH5*-mediated YAP m6A modification were also observed [168]. One study showed that *YTHDC2* expression was higher in late-stage ovaries, and that the disruption of *YTHDC2* expression in mice led to impaired meiosis and an ovarian insufficiency phenotype [169]. *TRDMT1*, an RNA m5C methyltransferase, regulated POF by repairing reactive oxygen species-triggered DNA damage in GCs. Hence, in *TRDMT1* mutants (TRDMT1^G155V^), DNA repair was severely impaired after reactive oxygen species-induced damage, leading to increased RNA m5C methylation activity [170].

There are few studies on RNA modifications in POF, and most current studies are limited to m6A or m5A modifications, the remaining types of modifications need to be explored further. In addition, relevant individual RNAs have not been explored deeply. RNA modification assumes a pivotal role in ovarian germ cell development and function. Aberrant RNA modification during oocyte and egg formation potentially leads to diminished egg quality, thereby impacting fertility. On one hand, it may hinder the success rates of in vitro fertilization and other assisted reproductive techniques. On the other hand, these modification abnormalities can impair egg development, potentially compromising embryo quality and stability, thereby elevating the risk of miscarriage and reducing successful pregnancy rates. Additionally, RNA modifications are believed to influence ovarian function by regulating several hormones intricately associated with follicular development and their respective signaling pathways. As an instance, the mRNA of the progesterone receptor (PGR) underwent m6A modification, thereby bolstering PGR protein translation efficiency in a YTHDF1-dependent manner[171]. The total m6A levels exhibited a significant decrease in the 4-Vinylcyclohexene diepoxide-treated mouse POF model, particularly in both preantral and antral follicles. Concurrently, there was an elevation in plasma follicle-stimulating hormone levels and a reduction in AMH levels[168]. In addition, RNA modification may serve as a potential therapeutic target as well as a potential biomarker for POF.

Therefore, future studies on POF and RNA modifications might concentrate on developing innovative therapeutic approaches centered on RNA modifications. This could involve the exploration of modulators targeting m6A, m5C, or other RNA modification types to potentially restore ovarian function or impede the progression of POF. Additionally, forthcoming investigations could delve into the role of RNA modifications in the early detection and treatment of POF, aiming to identify and validate specific RNA modifications as potential biomarkers for this condition.

### 5.5 Epigenetic modification-based treatment for POF

Cuscuta chinensis flavonoids regulated H19/Igf2 methylation to increase the levels of reproductive hormones and receptors and reduce apoptosis by upregulating the expression of DNMTs [172]. The allografting of brown adipose tissue improved DNA methylation, thus enhancing follicle and oocyte quality in mice [173].

Mesenchymal stromal cell-derived exosomes (MSCs-Exos) were known to contain several ncRNAs, and treatment with MSCs-Exos could reduce apoptosis and autophagy in POF. The expression of miRNAs and their target genes was associated with proliferation, angiogenesis, vasculogenesis, genomic integrity, and oocyte quality in cumulus cells [174]. miR-22-3p in exosomes derived from umbilical cord MSCs (hUCMSC-Exos) attenuated the apoptosis of mouse ovarian GCs (OGCs) and could improve ovarian function in a mouse model of POF by targeting the KLF6 and ATF4-ATF3-CHOP pathways [175]. Further, miR-644-5p and miR-144-5p both reduced apoptosis in GCs by targeting p53 and PTEN, respectively [176, 177]. Meanwhile, miR-126-3p acted on the PI3K/AKT/mTOR signaling pathway to promote angiogenesis and attenuate the apoptosis of OGCs in POF [178]. Further, both miR-126-3p and miR-21 downregulated LATS1, which reduced the levels of phosphorylated LOXL2 and YAP and ultimately promoted estrogen secretion in OGCs [178, 179]. Interestingly, circLRRC-8A in MSCs-Exos could protect senescent cells by mitigating oxidative damage via the circLRRC8A/miR-125a-3p/NFE2L1 axis [180]. Moreover, miR-369-3p in human amniotic fluid stem cell-derived exosomes (HuAFSC) specifically downregulated YAF2 expression, inhibited PDCD5/p53 stabilization, and reduced apoptosis in OGCs [181]. In addition, lncRNA-DANCR bound to hNRNPC and p53 to regulate hNRNPC-p53 interactions, thereby inhibiting p53-dependent GCs aging[182]. Furthermore, lncRNA nuclear enriched abundant transcript 1 (NEAT1) reduced miR-654 expression and modulated the STC2/MAPK pathway to reduce apoptosis and autophagy in POF [183]. Notably, melatonin inhibited miR-15a-5p and activated the Stat3 and PI3K-Akt-mTOR pathways to block GCs autophagy [184]. And miR-99a-5p in macrophage-secreted extracellular vesicles ameliorated the inflammatory microenvironment within ovaries via the PI3K/mTOR signaling pathway [185]. Juvenile rhesus monkey bone marrow mesenchymal stem cells could upregulate FTO protein expression and downregulate overall m6A levels, thereby reversing GCs aging [186]. The reagents or methods for the treatment of POF and their therapeutic mechanisms and effects were summarized in Table 6.

**Table 6:** Processing reagents or methods in POF and their therapeutic mechanisms and effects.

Treatment reagents or methods	Molecular mechanism	Treatment effect	References
**Cuscuta chinensis flavonoids**	Upregulation of DNMT expression to regulate H19/Igf2 methylation	Increasing levels of reproductive hormones and receptors and decreased apoptosis	[172]
**Allogeneic transplantation of brown adipose tissue**	Improving methylation	Improving follicle and oocyte quality	[173]
**miR-22-3p**	Targeting KLF6 and ATF4-ATF3-CHOP pathways	Attenuating OGCs apoptosis and improving ovarian function	[175]
**miR-644-5p**	Targeting p53	Reducing apoptosis of GC	[176]
**miR-144-5p**	Targeting PTEN	Reducing apoptosis of GC	[177]
**miR-126-3p**	Targeting sequences in the 3' untranslated region of PIK3R2 in OGCs.	Promoting angiogenesis and reduce apoptosis of OGCs in POF	[178]
**miR-21**	Reducing phosphorylation of LOXL2 and YAP	Promoting estrogen secretion	[179]
**CircLRRC-8A**	Treating oxidative damage through circLRRC8A/miR-125a-3p/NFE2L1 axis	Protecting aging cells	[180]
**miR-369-3p**	Lowering YAF2 and inhibiting the stability of PDCD5/p53	Reducing apoptosis of OGCs	[181]
**LncRNA**	Regulating the hNRNPC-p53 interaction	Inhibiting OGCs senescence	[182]
**LncRNA**	Reducing miR-654 and regulating the STC2/MAPK pathway	Reducing apoptosis and autophagy	[183]
**melatonin**	Inhibiting miR-15a-5p and activating Stat3 and PI3K-Akt mTOR pathways	Blocking OGCs autophagy	[184]
**miR-99a-5p**	Participating in the PI3K/mTOR signaling pathway	Improving the inflammatory microenvironment in the ovaries	[185]
**Bone marrow mesenchymal stromal cells**	Increasing FTO proteins and decreasing overall levels of m6A	Reversing GC Aging	[186]

The aforementioned studies collectively highlighted diverse therapeutic pathways and molecular mechanisms crucial in regulating ovarian function. They encompassed processes aimed at reducing apoptosis, enhancing oocyte quality, influencing angiogenesis, mitigating oxidative damage, and inhibiting autophagy. These findings significantly contribute to novel insights and directions in the research of reproductive disorders and potential treatments. Epigenetic therapy shows promise in tackling fertility disorders triggered by age-related alterations in reproductive cells. Moreover, it has the potential to alleviate the psychological burden caused by delayed reproduction due to age-related changes in these cells. Utilizing epigenetic-based treatment approaches is expected to facilitate personalized interventions tailored to individual genetic backgrounds and specific epigenetic modification patterns. Future research may delve into developing pharmaceuticals grounded in epigenetics, striving for more accessible treatments to overcome limitations linked to conventional therapies.

However, epigenetic therapies encounter several challenges, primarily encompassing the following aspects. Firstly, technical hurdles arise due to the intricate nature of epigenetic regulatory mechanisms, posing difficulty in devising therapies tailored to specific diseases. Secondly, safety concerns emerge, wherein therapies designed to target abnormal tissues and cells may inadvertently impact normal tissues and organs. Ensuring the safety and non-toxicity of these therapies is paramount. Ethical considerations also loom large. The development of novel epigenetic therapies carries potential risks that could influence the genome and cellular structure of individuals. The assessment of potential risks and side effects must be meticulous. Hence, stringent legal regulations are imperative throughout the development and application phases to guarantee the legality and safety of these treatments.

## 6. Conclusion

Germ cell senescence involves a complex interplay of genetic, environmental, autoimmune, lifestyle, and age-related factors. In contemporary society, there's a steady rise in the reproductive age for both men and women, attributed to economic advancements and shifts in societal norms. However, this extension in reproductive years, coupled with occurrences like female POF, contributes to a decline in germ cell quality. This decline leads to significant physical and psychological stress, potentially impacting the health of future generations. In recent years, significant attention has been devoted to studying the epigenetic mechanisms underlying germ cell senescence, spurred by technological breakthroughs and heightened interest. This increased focus has led to advancements in understanding and exploring therapeutic interventions to address the challenges posed by declining germ cell quality. However, although a clear correlation between germ cell senescence and epigenetic inheritance has been identified, little is known about the underlying mechanisms, especially in spermatozoa. Future studies must thus focus on the specific mechanisms of epigenetic regulation. In terms of therapeutic approaches, although epigenetic inheritance is strongly associated with germ cell senescence, some studies indicate that it may be possible to delay or even reverse germ cell senescence through epigenetic interventions. However, specific epigenetic drugs are yet to be developed. An in-depth investigation into the epigenetic regulation of germ cell senescence and anti-senescence epigenetic interventions can provide a theoretical basis for the development of new therapeutic strategies, which could improve fertility and enhance human reproductive health.

Exploring the ongoing relationship between epigenetic modifications and germ cell senescence encompasses several pivotal aspects. Primarily, the intricate mechanisms linking epigenetic modifications to germ cell senescence demand further elucidation. Current studies on DNA methylation, ncRNA regulation, histone modification, chromatin remodeling, and RNA modification often remain compartmentalized. To comprehensively grasp how gene expression is governed within germ cell senescence, future investigations should adopt a holistic approach towards the entire epigenome. Secondly, the close correlation of germ cell aging with various age-related disorders, such as infertility, chromosomal anomalies, and pregnancy complications, prompts deeper exploration into the contribution of epigenetic modifications to these conditions. A more profound understanding in this realm could open avenues for novel treatment modalities and preventative strategies. Technological advancements present a pivotal facet. Progressing towards higher-resolution, precise, user-friendly, and cost-effective detection techniques and tools will facilitate a more exhaustive and detailed examination of the correlation between epigenetic modifications and germ cell aging. Enhanced technological capabilities will enable the identification of subtle modification changes and a more profound exploration of their impact on germ cell functionality and overall health. Looking ahead, interdisciplinary collaborations and in-depth exploration are anticipated to offer insights into these inquiries, illuminating the intricate connection between epigenetic modifications and germ cell aging.

## Data Availability

All data generated or analyzed during this study are included in this published article.
